# Regulation of innate immune signaling by IRAK proteins

**DOI:** 10.3389/fimmu.2023.1133354

**Published:** 2023-02-14

**Authors:** Milton Pereira, Ricardo T. Gazzinelli

**Affiliations:** ^1^ Division of Infectious Diseases and Immunology, Department of Medicine, University of Massachusetts Chan Medical School, Worcester, MA, United States; ^2^ Centro de Tecnologia de Vacinas, Universidade Federal de Minas Gerais, Belo Horizonte, MG, Brazil; ^3^ Instituto René Rachou, Fundação Oswaldo Cruz, Belo Horizonte, MG, Brazil; ^4^ Plataforma de Medicina Translacional, Fundação Oswaldo Cruz, Ribeirão Preto, SP, Brazil

**Keywords:** TLR, IRAK, innate immunity, cell signaling, IL-1R, inflammation

## Abstract

The Toll-like receptors (TLRs) and interleukin-1 receptors (IL-1R) families are of paramount importance in coordinating the early immune response to pathogens. Signaling *via* most TLRs and IL-1Rs is mediated by the protein myeloid differentiation primary-response protein 88 (MyD88). This signaling adaptor forms the scaffold of the myddosome, a molecular platform that employs IL-1R-associated kinase (IRAK) proteins as main players for transducing signals. These kinases are essential in controlling gene transcription by regulating myddosome assembly, stability, activity and disassembly. Additionally, IRAKs play key roles in other biologically relevant responses such as inflammasome formation and immunometabolism. Here, we summarize some of the key aspects of IRAK biology in innate immunity.

## 1 Introduction

Pattern recognition receptors (PRRs) such as Toll-like receptors (TLRs) and nucleotide oligomerization domain-like receptors (NLRs) are of paramount importance in the innate host resistance to microbial infections. These receptors recognize pathogen-associated molecular patterns (PAMPs) and danger-associated molecular patterns (DAMPs), transducing these signals into biological responses. TLRs accomplishes this by recruiting the signaling adaptors myeloid differentiation primary-response protein 88 (MyD88) and/or TIR domain-containing adaptor protein inducing IFN-β (TRIF) and their respective co-adaptors MyD88-adapter-like (Mal) and TRIF-related adaptor molecule (TRAM) (1–8). Most TLRs employ MyD88 as signaling adaptor, with the exceptions being TLR3 that signals exclusively *via* TRIF and TLR4 that uses both TRIF and MyD88 (2). In addition to PRRs, many of the early inflammatory responses are modulated by the interleukin (IL)-1 family of cytokines, which include IL-1α, IL-1β, IL-18 and IL-33 (9). Responses to these cytokines are mediated by the IL-1 receptor (IL-1R) and the closely related IL-18R and IL-33R, all of which employs MyD88 as signaling adaptor, similarly to TLRs (9–11).

Engagement of IL-1R or most TLRs results in the hierarchical recruitment of MyD88, IL-1 receptor-associated kinase (IRAK) 4, and IRAK2, or alternatively IRAK1, followed by E3 ubiquitin ligase TNF receptor associated factor 6 (TRAF6) (10–18), forming a supramolecular organizing center (SMOC) termed myddosome (14, 19). This SMOC controls the inflammatory response by mediating transcriptional and non-transcriptional events. These include regulation of transcription factors, control of post-transcriptional mRNA stability and transport, metabolic responses such as glycolysis, oxidative phosphorylation, and inflammasome activation (20–26).

**Figure 1:**
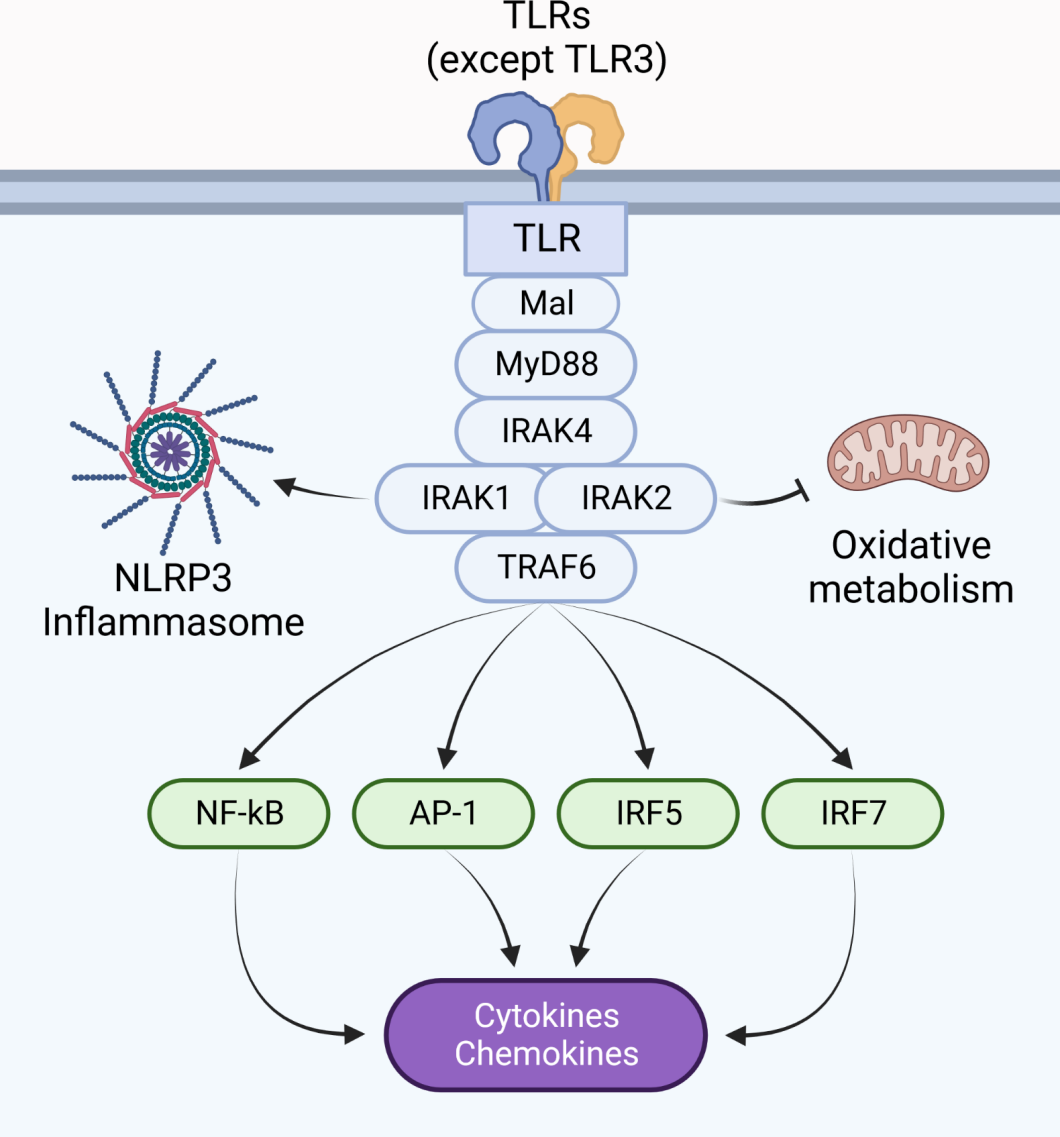
Pro-inflammatory responses are coordinated by the myddosome. The myddosome is a supramolecular signaling complex assembled upon dimerization of IL-1Rs and most TLRs (except TLR3), and is composed of MyD88, IRAK4, IRAK1 and/or IRAK2, in addition to TRAF6. Its activity coordinates the inflammatory response by activating NLRs, modulating metabolic responses, and regulating gene transcription *via* activation of transcription factors such as NF-κB, AP-1, IRF5, and IRF7.

Activation of the ubiquitin ligase TRAF6, in addition to ubiquitin ligases of the Pellino family (17, 27–32), are tightly regulated by upstream IRAK proteins. This event controls the activation of mitogen-activated protein kinases (MAPK), leading to transcription of activation protein 1 (AP-1) target genes, in addition to activation of IκB kinase complex, responsible for activation of transcription factors such as nuclear factor kappa B (NF-κB), interferon regulatory factor (IRF) 5 and IRF7 (8, 33–40) (**Figure 1**). This review will discuss how IRAK proteins regulate myddosome signaling: from assembly to disassembly, including stability, stimulation and inhibition of transcriptional and non-transcriptional responses.

## 2 Regulation of cell signaling by the IRAK family

IRAK proteins are serine/threonine kinases originally discovered in the context of IL-1R signaling (10, 12, 18, 41–45). Their recruitment to IL-1Rs is mediated by the signaling adaptor MyD88 (11), which is also shared by most TLRs (except TLR3) (2, 8). These proteins act as the main regulators of myddosome activity and are required for controlling a wide range of infections (12, 46–48). As such, mutations in these proteins are often associated with enhanced susceptibility to infections, sepsis, sterile inflammation, cancer (48–54), etc., and many pathogens have evolved evasion mechanisms that interfere with IRAKs (55–60).

### 2.1 The structure of IRAK proteins

**Figure 2:**
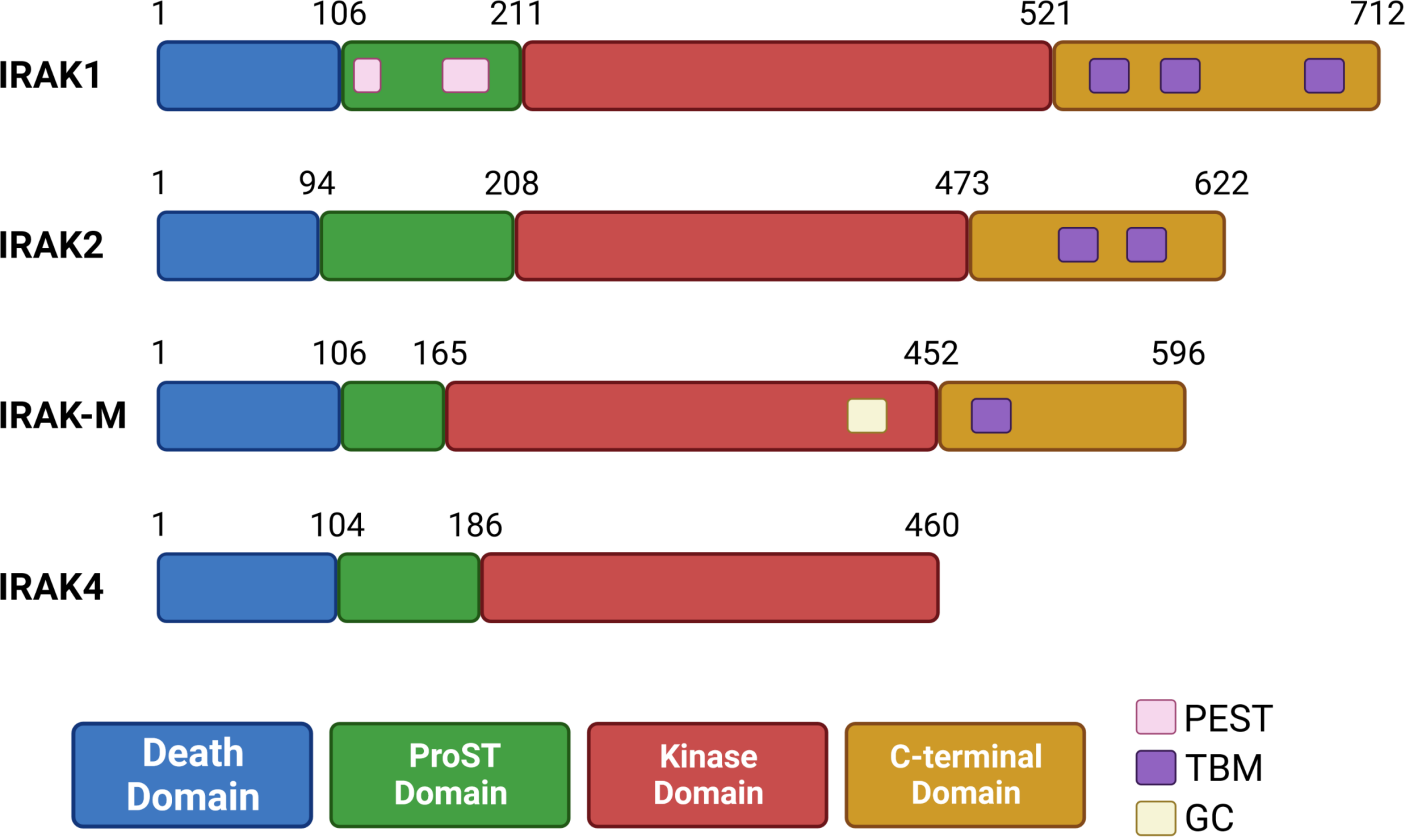
Organization of human IRAK proteins. IRAK proteins contain a death domain (DD), ProST domain, kinase domain (inactive in IRAK-M) and a C-terminal domain (absent in IRAK4). IRAK1 has two putative PEST sequences in its ProST region. IRAK-M contains a guanylate cyclase (GC) center in its kinase domain. The C-terminal domain contains up to three TRAF6 binding motifs (TBMs), and is absent in IRAK4.

IRAKs are highly conserved in vertebrates and contain a N-terminal death domain (DD), a ProST domain, a kinase domain (KD), and finally a C-terminal domain containing TRAF6 binding motifs (TBMs) (61, 62). DDs present in IRAKs are involved in homotypic protein-protein interactions, such as the interactions between IRAK4 and MyD88 discussed below. ProST domains (rich in proline, serine and threonine residues) are targeted by autophosphorylation, and are involved in activation of IRAK proteins. Close to its ProST domain, IRAK1 may also contain two PEST domains (63, 64) (rich in proline (P), glutamate (E) or aspartic acid, serine (S), and threonine (T)) which usually target proteins for degradation (65). All IRAKs contain a kinase domain, which is inactive in IRAK-M, due absence of a key aspartate residue (66). IRAK2 also lacks this aspartate residue, but this protein has kinase activity and behaves like an atypical kinase, discussed below (67). Interestingly, IRAK-M contains an active guanylate cyclase (GC) center in its kinase domain (68, 69). Finally, the C-terminal domain contains TBMs: three in IRAK1, two in IRAK2, and one in IRAK-M. IRAK4 does not contain a C-terminal domain and has no TBMs (62, 70) (**Figure 2**).

**Figure 3:**
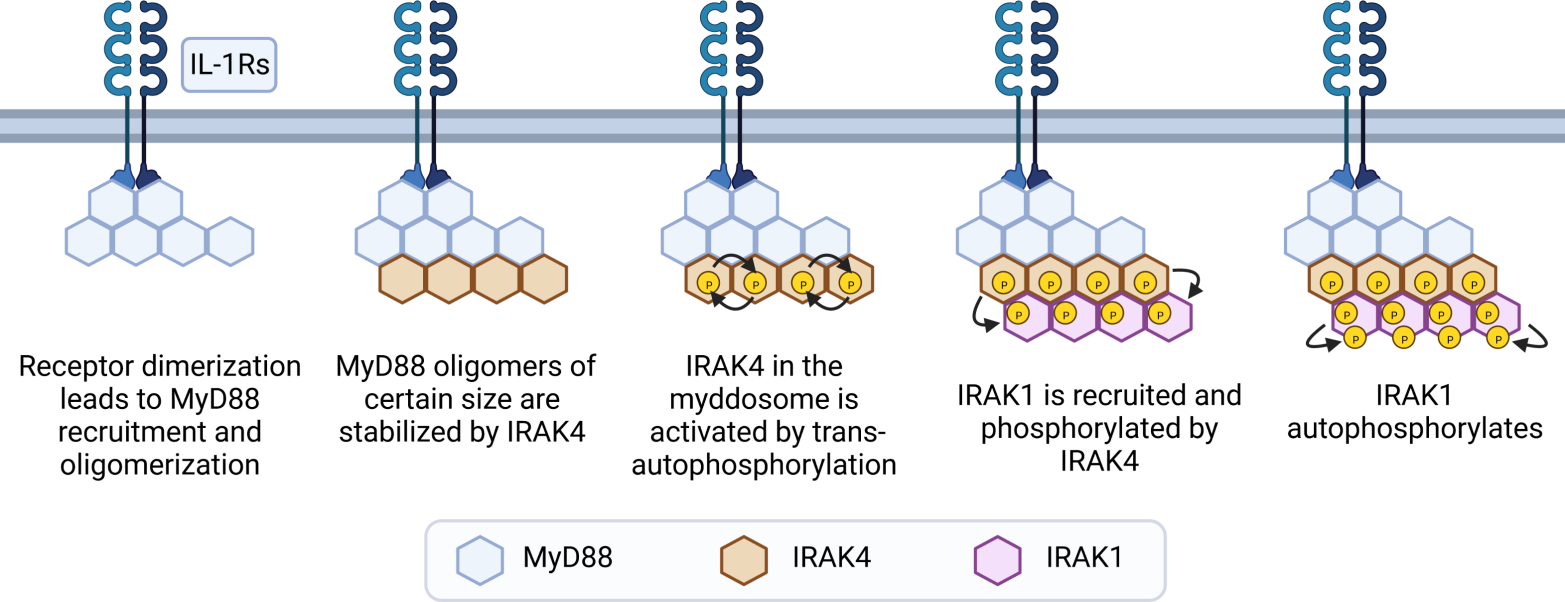
Steps of myddosome assembly. Dimerization of IL-1R or TLRs (except TLR3) leads to conformational changes in their cytoplasmic TIR domains, allowing TIR-TIR interactions with molecules of MyD88 to occur, either directly or *via* Mal dimers. This leads to formation of small and unstable MyD88 oligomers, which grow if receptor activation is sustained. After reaching a certain size threshold, MyD88 oligomers are stabilized by IRAK4. Interactions with MyD88 allows IRAK4 to trans-autophosphorylate and recruit IRAK1. Next, IRAK1 is activated by phosphorylation events mediated by IRAK4, and then further activated by auto-phosphorylation in its ProST regions, allowing signaling to occur.

Recognition of its ligand results in receptor dimerization followed by conformational changes in their cytoplasmic Toll/IL-1 receptor (TIR) domains. These conformational changes allow the receptor TIR domains to interact with 6 to 8 molecules of MyD88 *via* TIR-TIR homotypic interactions directly or *via* 2 molecules of Mal (also named TIR domain containing adaptor protein, TIRAP). Next, the MyD88 DDs interact with IRAK4’s DDs, in a stoichiometry of 6 to 8 molecules of MyD88 to 4 molecules of IRAK4. This stable interaction allows IRAK2 molecules (and presumably IRAK1) to interact with IRAK4 in a stoichiometry of 4:4. This forms a three-layered structure, arranged as a single-stranded left-handed helix. The proximity of multiple IRAK4 and downstream IRAKs 1 and 2 allows for sequential activation of their kinase activities to occur (15, 71, 72). IRAK4 molecules bound to MyD88 can auto trans-autophosphorylate, which then recruits IRAK1 to the myddosome and phosphorylates it (44, 73). This initial phosphorylation step activates IRAK1 and stimulates auto-hyperphosphorylation in its ProST region (rich in proline, serine and threonine residues) (**Figure 3**) (64). In resting cells, Toll-interacting protein (Tollip) interacts with the DD and kinase domain of IRAK1 (and presumably IRAK2), keeping these kinases in an inhibited state (74, 75). Upon receptor activation, PTEN induced protein kinase 1 (PINK1) interacts with Tollip/IRAK1 dimers, facilitating the delivery of IRAK1 to the myddosome (76).

The details involved in IRAK2 activation are less clear, but it is generally assumed to be similar to IRAK1. Initially, IRAK2 was thought to be a pseudokinase due its low *in vitro* kinase activity and substitution of a key aspartate residue to asparagine in its kinase domain (45), but later studies rebutted this claim and demonstrated that IRAK2 does possess kinase activity, which is activated by IRAK4-mediated phosphorylation on its lysine 237. Accordingly, point-mutated IRAK2 K237A fails to induce cytokine production, and no IRAK2 phosphorylation is observed in kinase-deficient IRAK4 macrophages (67, 77). Instead of a pseudokinase, IRAK2 can be classified as an atypical kinase, due substitution of residues 333 (Asp to Asn) and 351 (Asp to His) in its kinase domain (24, 78). Following activation by IRAK4, IRAK2 is autophosphorylated on residues S136 and T140 (24, 79). These autophosphorylation events are involved in IRAK2 translocation to the nuclei and to the mitochondria, where IRAK2 can act as a transporter of mRNAs to the cytoplasm and as an inhibitor of oxidative phosphorylation, respectively (24, 26). It is unclear whether these are the same autophosphorylation events necessary for NF-κB and MAPK activation.

**Figure 4:**
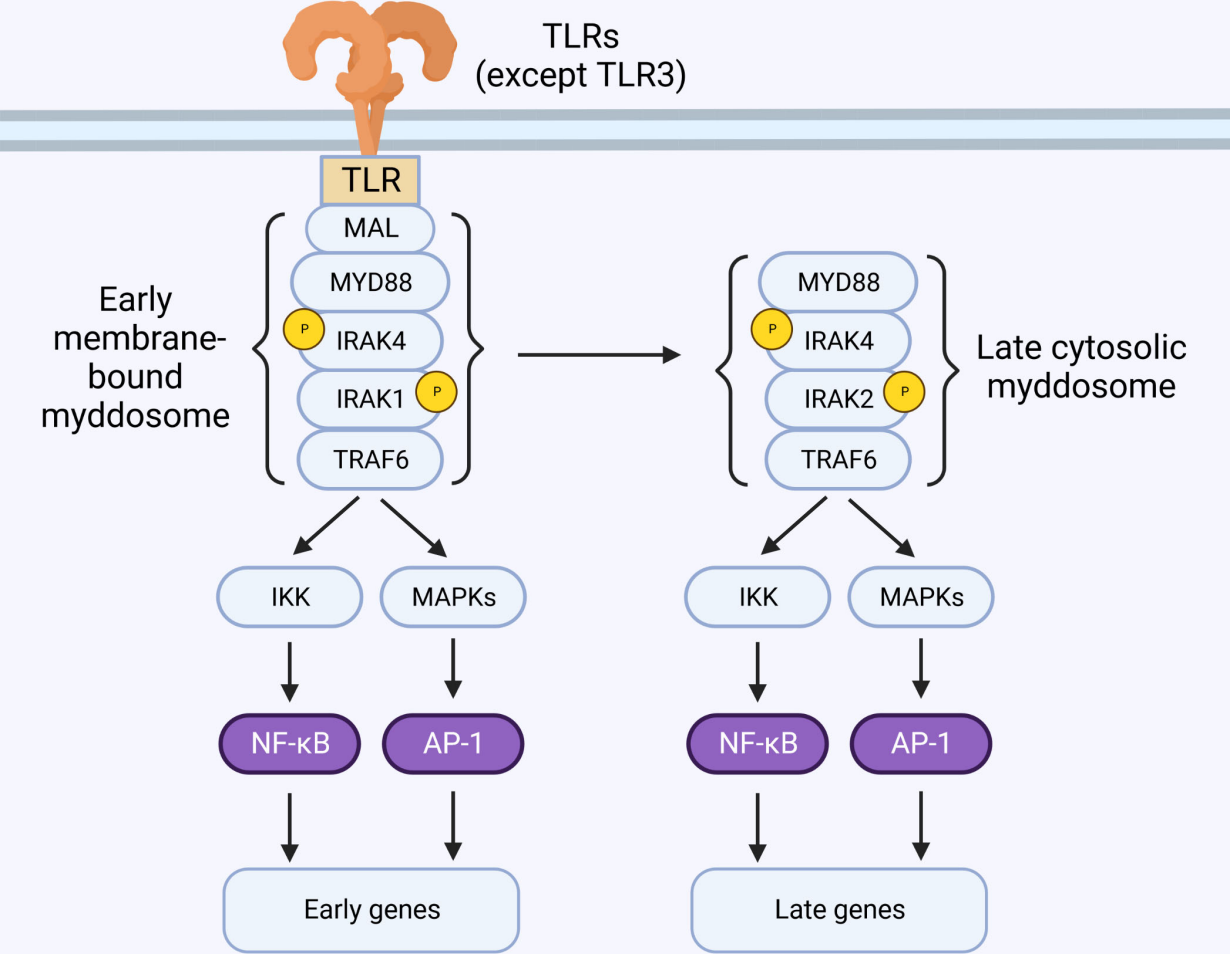
IRAK2 is essential for late-phase TLR signaling. TLR activation at the membrane triggers the sequential recruitment of Mal, MyD88, IRAK4, IRAK1 and TRAF6, forming the myddosome. These early membrane-bound complexes are short-lived and detectable only within minutes of stimulation. Sustained TLR stimulation leads to formation of cytosolic myddosomes containing IRAK2.

The model of myddosome formation suggested above implies that either IRAK1 or IRAK2 can be recruited by IRAK4. In reality, the details about which IRAK is recruited to the myddosome are largely unknown. Immunoprecipitation experiments from murine macrophages suggests that IRAK2 is the main protein recruited to the myddosome, and IRAK1 is only detectable bound to the myddosome at early time-points and in situations where IRAK4 kinase activity is deficient (77, 80, 81). This could be a consequence of IRAK1 degradation (which requires IRAK4 kinase activity (80–83) and suggests that IRAK1 is recruited to early myddosomes, while later complexes contain IRAK2. This model is consistent with the observation that IRAK1 and IRAK2 are redundant at early time-points, but IRAK2 is required for later responses (**Figure 4**) (67). However, other cell types require IRAK1 (43). The molecular basis for this preference is unknown but may involve differences in IRAK1 and IRAK2 expression levels, half-life, splice variants, and regulation by additional molecules.

Via their C-terminal TBM, activated IRAK1 or IRAK2 can interact and activate TRAF6 (17, 84–86). IRAK2 binding to TRAF6 requires its E528 residue (mouse E525) (84, 87), and full activation requires its last 55 amino acids (88), while IRAK1 binding requires either of its two TBMs (70). Although the molecular mechanism of how IRAKs 1 and 2 activate TRAF6 are not completely understood, evidence suggests that IRAK-TRAF6 interactions results in TRAF6 auto-activation *via* auto-K63-ubiquitination (89), followed by its translocation from the myddosome to the cytosol (86), where it catalyzes the K63-ubiquitination of various targets such as IκB kinase (IKK) and IRAK1 (90, 91).

Recently, the IRAK4 scaffold was implicated in TRIF-mediated TRAF6 activation (77), and previously it was found that IRAK4 and IRAK1 can physically interact with TRAM (92). IRAK4 lacks the C-terminal TBM, which is present in IRAKs 1 and 2 (85), thus making it possible that the TRIF-mediated TRAF6 activation requires either IRAK1 or IRAK2 in addition to IRAK4, in a MyD88-independent way (77). While experimental evidence is still required to confirm the existence of the triffosome, these data raise the possibility that IRAKs are part of the putative triffosome and can lead to TRAF6 activation in the TLR4-TRIF pathway.

## 3 Control of myddosome stability and termination of signal transduction

The myddosome is a multifunctional SMOC formed at the cell membrane within minutes of TLR or IL-1R stimulation, and its assembly is essential for regulating biological responses such as production of inflammatory cytokines and metabolic responses (16, 19). As such, tight regulation of myddosome formation, stability, and disassembly is of paramount importance. We find IRAK4 at the center of these tightly regulated processes.

### 3.1 MyD88 oligomerization and myddosome stability is controlled by IRAK4

Stimulation of IL-1R results in rapid formation of small and unstable MyD88-oligomers. Prolonged exposure to IL-1β, however, favors the formation of larger oligomers that are sensed by IRAK4 (80). This mechanism ensures that only *bona fide* receptor activation can propagate signal. Live-cell microscopy suggests that MyD88 signaling is required for sustained activation of transcription factors (93), but early membrane-bound complexes containing MyD88, IRAK4 and IRAK1 are short-lived and detectable on a scale of a few minutes after stimulation (80, 94). Interestingly, biochemical isolation of endogenous myddosomes are often accomplished at time-points of 30 minutes or more, and these complexes predominantly contain IRAK2 instead of IRAK1 (77, 81, 95). Evidence suggests that stable MyD88-containing helical complexes occur in the cytosol, and can recruit IRAK4 after stimulation (96). Indeed, transfection of MyD88^L265P^ (gain-of-function mutation) triggers myddosome formation and signaling in the cytosol, bypassing assembly in the membrane (97). Thus, it is feasible that early myddosome assembly in the membrane is a process regulated by IRAK4, and act as a nucleating step that results in the formation of stable signaling complexes in the cytosol, detectable by immunoprecipitation at later time-points (**Figure 4**) (25, 80, 94, 96, 98, 99).

Formation of longer MyD88 oligomers with enhanced stability is observed in IRAK4 knockout cells, while signal transduction is severely inhibited (12, 80). Similarly, IRAK4 inhibition or presence of kinase-dead IRAK4 increases myddosome stability, with severe deficiency in signal transduction. This is accomplished without increasing the size of MyD88 oligomers, as this activity is kinase-independent (80–82). Interestingly, IRAK4 inhibition does not affect IRAK2 recruitment to the myddosome, while recruitment of IRAK1 is in fact enhanced (25, 77, 80, 81, 98). In this case, neither IRAK1 or IRAK2 appear to be active (77), and the enhanced IRAK1 recruitment could be a consequence of enhanced myddosome stability and/or deficient IRAK1 degradation, normally triggered by IRAK4 kinase activity (64, 77, 83). Recruitment of IRAK4 to the myddosome leads to recruitment and phosphorylation of IRAK 1 and 2, turning on their autophosphorylation activities (67, 73, 100). In humans, however, IRAK1 can auto-phosphorylate when IRAK4 kinase activity is lost, and its activation likely involves an allosteric mechanism triggered by its interaction with IRAK4 in the myddosome (101, 102). No experimental evidence exists for similar compensatory mechanism involving IRAK2. Although in murine macrophages these IRAK4 kinase-deficient myddosomes fail to lead to cytokine production (77), it is possible that recruitment of IRAK1 and/or IRAK2 to this complex could compensate for the lack of IRAK4 kinase activity in other cell type or species (101, 103).

### 3.2 IRAK4 activation initiates a negative feedback loop

**Figure 5:**
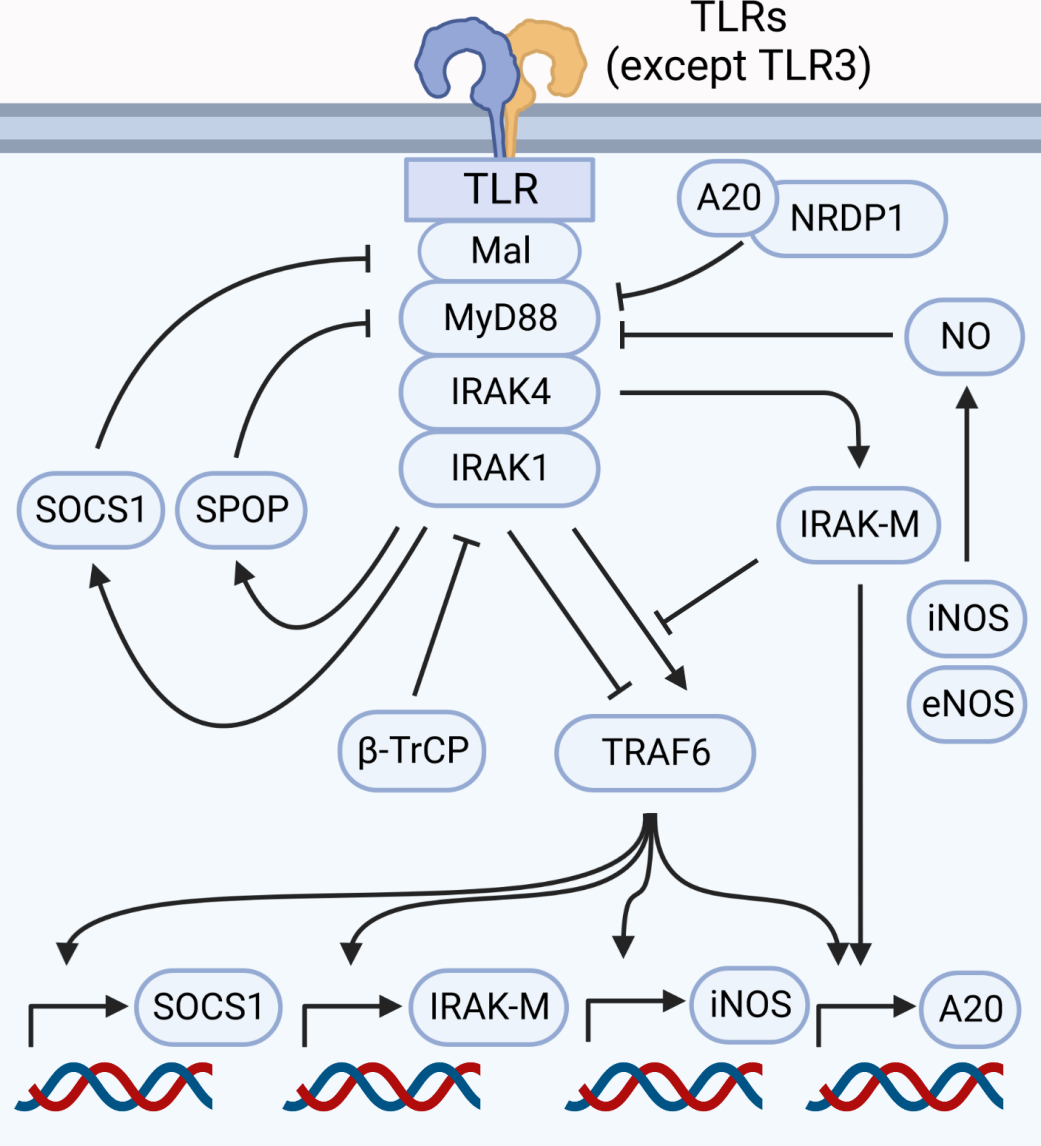
Myddosome signaling is regulated at nearly every step. Signaling through the myddosome leads to transcription of negative regulators such as SOCS1, iNOS, IRAK-M and A20. Proteasomal degradation of components such as Mal (controlled by SOCS1), MyD88 (controlled by A20 and NRDP1, in addition to SPOP in HSC), IRAK1 (controlled by β-TrCP), and TRAF6 (triggered by IRAK1) are likely involved in myddosome disassembly and signal termination. IRAK-M antagonizes the myddosome by inhibiting the binding between IRAK1 and TRAF6, besides stimulating A20 expression. Production of NO by eNOS and iNOS leads to S-Nitrosylation of MyD88, inhibiting signal transduction.

Myddosome formation leads to induction of pro-inflammatory and other adaptations, all of which are required for an efficient response. However, uncontrolled myddosome activation is detrimental, as evidenced by gain-of-function mutations linked to diseases (104, 105). Thus, termination of the response can be as important as its initiation. IRAK4 activity is central to the myddosome-mediated pro-inflammatory responses, but it also initiates a series of adaptations that inhibits myddosome signaling, forming a negative feedback loop. Amongst these adaptations are induction of antagonists such as IRAK-M and A20, post-translational modifications such as S-Nitrosylation, and the degradation of myddosome components such as Mal, IRAK1, TRAF6 and MyD88 itself (64, 66, 83, 106–109). This ensures tight regulation of the pro-inflammatory response initiated by TLRs and IL-1Rs (**Figure 5**).

Study of neuregulin receptor degradation protein-1 (NRDP1) revealed that this E3 ubiquitin-ligase modulates cytokine production *via* K48 ubiquitination of MyD88 and K63 ubiquitination of tank binding kinase 1 (TBK1). This results in production of type I interferons due TBK1 activation, and inhibition of pro-inflammatory cytokines due proteasomal degradation of MyD88 (108). This MyD88 ubiquitination requires physical interactions between myddosome components, NRDP1 and A20 (108, 110). Although no direct links between NRDP1 and IRAKs were established, A20 expression is induced by NF-κB (111), suggesting that MyD88 degradation requires a functional myddosome capable of driving A20 expression. Additionally, other molecules were linked to MyD88 ubiquitination and degradation (112, 113), including Speckle-type BTB–POZ protein (SPOP) (114, 115). SPOP is a ubiquitin ligase highly expressed in hematopoietic stem cells (HSC), and while the details on how SPOP regulates MyD88 degradation are largely unknown, evidence suggests that IRAK4 is required for SPOP-mediated K48 ubiquitination of MyD88 (114).

TLR4 activation leads to ubiquitination and degradation of the co-adaptor Mal, likely playing a role in termination of signal and myddosome disassembly (106). Experiments involving overexpression or pharmacological inhibition of IRAK1 and IRAK4 demonstrates that these IRAKs are required for Mal phosphorylation and subsequent ubiquitination and degradation (106). This is likely mediated by suppressor of cytokine signaling (SOCS) 1 and 3, as evidence suggests that upon TLR activation SOCS1 (and potentially SOCS3) ubiquitinate Mal leading to its proteasomal degradation and suppression of cytokine production (116–118). While Mal degradation is observed within 15 to 30 minutes of TLR2 or TLR4 activation (117), TLR-dependent production of nitric oxide (NO) can upregulate SOCS1 expression, contributing to hypo-responsiveness upon long lipopolysaccharide (LPS) stimulations (119). High concentrations of NO produced by either endothelial nitric oxide synthase (eNOS) or inducible nitric oxide synthases (iNOS) can directly inhibit myddosome signaling, due nitrosylation of cysteine residues in the MyD88 protein (109).

IRAK1 activity rapidly decreases after TLR stimulation, which correlates with its degradation. Interestingly, IRAK2 is not degraded and can sustain signaling for longer (67, 77). One proposed mechanism for this behavior involves the presence of two putative PEST sequences in IRAK1 (ranging from amino acids 117 to 133 and 153 to 180), which are absent in IRAK2 (63, 64). It was suggested that IRAK4 kinase activity triggers hyperphosphorylation in or adjacent to the IRAK1 PEST sequences, targeting it for degradation (64, 83). It is presently unclear whether the proteasome is responsible for IRAK1 degradation: while some reports suggests that IRAK1 is K48-ubiquitinated by β-transducin repeats-containing proteins (β-TrCP) and degraded by the proteasome following IL-1R or TLR stimulation (120–122), other studies suggest that IRAK1 is mainly targeted by K63-ubiquitination and its degradation is proteasome-independent (83, 123). Whatever the case may be, the PEST-hypothesis cannot be ruled-out, as PEST-containing proteins can also be targeted by other proteases such as calpain (65) and functional validation of PEST regions in IRAK proteins is still required.

The E3-ubiquitin ligase TRAF6 is a key mediator of TLR and IL-1R responses, and downregulation of its expression is one of the reported mechanisms involved in termination of MyD88-dependent responses. TRAF6 expression levels decreases after long periods of TLR activation (around 24 hours *in vitro*) due proteasomal degradation. The presence of IRAK1, but not its kinase activity, is involved in downregulating TRAF6. One suggested mechanism is that IRAK1 bound to TRAF6 is K48 and K63 ubiquitinated, and this interaction directs both proteins to the proteasome (107). Accordingly, mutations on IRAK1’s TBM can inhibit TRAF6 degradation. IRAK1 contains three C-terminal TBMs, and while deletion of all TBMs fail to transduce signal and induce cytokine production due deficient TRAF6 interaction, TRAF6 degradation, however, requires the DD in addition to one TBM (70). This suggests that depending on context IRAK1 can induce TRAF6 activation or degradation, but the molecular mechanisms for these antagonic activities are presently unknown.

In resting cells, Tollip is bound to IRAK1, supressing its activity (74, 75). Upon stimulation, Tollip releases IRAK1, which is then recruited to the myddosome. Interestingly, Tollip itself is a target of IRAK1-mediated phosphorylation (75), and is involved in the degradation of IL-1R and signal termination (124). It is presently unclear if Tollip phosphorylation triggers IL-1R degradation, but it is tempting to speculate that release and phosphorylation of Tollip is another negative feedback mechanism.

### 3.3 Myddosome inhibition by IRAK-M

While IRAKs 4, 1 and 2 are, in most conditions, responsible for amplifying the signal from activated TLRs or IL-1Rs, the pseudokinase IRAK-M (also known as IRAK3) is a negative regulator of this response (66). In humans, IRAK-M expression is restricted to cells of the myeloid lineage (45), but murine IRAK-M is detectable in a wider range of cell types (125). In macrophages, IRAK-M represses NF-κB and MAPK by a variety of mechanism, such as inhibition of the interaction between IRAK4 and IRAK1 with TRAF6 (66), stabilization of MAPK phosphatase 1 (MKP-1, a phosphatase responsible for inactivating the MAPK p38), and transcription of inhibitory molecules (66, 126, 127).

Despite negligible kinase activity, IRAK-M can induce a specific subset of NF-κB target-genes, which collectively inhibit the inflammatory response. Indeed, overexpression experiments originally described IRAK-M as a positive regulator of NF-κB signaling, not unlike IRAK1 and IRAK2 (45). Evidence suggests that in physiological conditions, IRAK-M can interact with MyD88 and IRAK4, forming a myddosome that activates transforming growth factor-β-activated kinase 1 (TAK1) and stimulates inhibitory genes such as A20 and NF-κB inhibitor-α (IκB-α) (127). Surprisingly, IRAK-M can also, in specific contexts, induce the expression of pro-inflammatory genes. Upon IL-33 stimulation of murine dendritic cells, the prolyl cis-trans isomerase PIN1 interacts with IRAK-M, promoting its nuclear translocation and transcription of genes involved in type 2 immunity and airway inflammation such as *Il6*, *Csf3*, *Cxcl2* and *Ccl5* (128). It is unclear how the pseudokinase IRAK-M activates gene transcription, but one intriguing possibility involves cGMP as second messenger: bioinformatics studies suggested that IRAK-M possess guanylate-cyclase activity (68), which was later experimentally confirmed (69). Transduction experiments demonstrates that wild type IRAK-M attenuates LPS-induced NF-κB activity, but a IRAK-M mutant with impaired guanylate cyclase activity does not (69). The details regarding this pathway are currently unknown, and further studies are required to confirm its importance in physiological conditions.

Notably, TLR activation leads to a NF-κB-dependent IRAK-M upregulation, forming a negative feedback loop that contributes to signal termination and tolerance (66, 129). This is not exclusive to TLRs and IL-1Rs, and is likely part of a more general mechanism for resolving inflammation, as IRAK-M can also be induced by other transcription factors including glucocorticoid receptors (130, 131). Due its generally anti-inflammatory activity, the induction of IRAK-M can be exploited by different pathogens as an evasion mechanism (59, 132).

## 4 IRAK4 scaffold and kinase activities

While IRAK4 is the main regulator of myddosome activity, its roles can be divided into “scaffold activity” and “kinase activity”, as suggested by various studies where cells expressing kinase-deficient IRAK4 do not phenocopy IRAK4 knockout cells (77, 133–135). Another surprising observation is that the IRAK4 scaffold, but not its kinase activity, is required for the production of pro-inflammatory cytokines in TLR-stimulated human cells (136). Understanding the subtle differences between IRAK4 scaffold and kinase activities is relevant from a medical standpoint, as IRAK4 inhibitors and degraders are currently under investigation (137).

IRAK4 is a key component of the myddosome, and it is believed that loss of IRAK4 or inhibition of its kinase activity phenocopies the loss of MyD88 (12, 46–49, 77, 133–135, 138). This is, however, context-dependent, as there are important differences between the IRAK4 scaffold and kinase activities depending on stimuli, cell type, species, etc. Kinase-deficient IRAK4 murine macrophages fail to produce pro-inflammatory cytokines upon stimulation of IL-1R and TLRs 2, 7, and 9 (77, 133–135). Interestingly, inhibition of IRAK4 kinase activity in human macrophages or fibroblasts does not impact cytokine production (101, 136). In murine macrophages inhibition of IRAK4 kinase activity impacts NF-κB and MAPK activation (77, 133–135), while in human monocytes this inhibition has no impact on NF-κB and MAPK, and only IRF5 activation is impaired (139).

In TLR4-stimulated mouse macrophages, inhibition of IRAK4 kinase activity does not completely impairs cytokine production, while loss of IRAK4 scaffold does (77, 133–135). This is likely due the fact that TLR4 signals *via* MyD88 and TRIF, and suggests that the putative triffosome employs the IRAK4 scaffold to activate TRAF6, which is the hub that links MyD88 and TRIF in TLR4 signaling (17, 77, 140). Although physical interactions between TRAM, TRAF6, IRAK4 and IRAK1 were previously described (92, 141), little is known on how the triffosome is assembled and how it activates TRAF6.

In the absence of IRAK4 kinase activity, an enhancement in IRAK1 recruitment is observed as long as the IRAK4 scaffold is present (77, 81). Previous studies suggest IRAK1 can bypass the initial phosphorylation performed by IRAK4, in situations where IRAK4 kinase activity is deficient (64, 102). While this is unlikely the case in murine macrophages, this compensatory mechanism could play a role in human fibroblasts (101, 103, 136, 142). This is further suggested by the D329A mutant in human IRAK4. This mutant lacks kinase activity, but IL-1β-stimulated IRAK4^D329A^ fibroblasts has partial cytokine production likely caused by the decreased interaction between IRAK4 and IRAK1, and not by the lack of IRAK4 kinase activity (82). This compensatory mechanism is not perfect, as IRAK4 kinase activity is required for IRAK1 degradation (83), suggesting that IRAK4 enzymatic activity controls both the initiation and termination of myddosome signaling.

## 5 IRAK1 and IRAK2 are not redundant in every context

Early studies on IL-1R demonstrated that the cytoplasmic fraction of this receptor physically interacts with a serine-threonine kinase responsible for signal transduction (41, 42). It was postulated at the time that a protein kinase termed IRAK was the responsible for this activity (42). Further study in human cells identified IRAK, a protein that shared high similarity to Pelle, responsible for activation of a NF-κB homolog in *Drosophila* (18). Generation of IRAK knockout mice, however, revealed that *in vivo* responses to IL-1β or IL-18 were only partially blocked, suggesting the existence of an alternative pathway (43). Additionally, *Irak1^-/-^* mice challenged with LPS showed only a subtle improvement in survival, further suggesting an alternative pathway (143). While these developments occurred, another Pelle-like protein termed IRAK2 was identified, also capable of interacting with IL-1R and the then discovered adaptor MyD88, and required for NF-κB activation (10). Development of IRAK2 knockout mice strongly suggested that this protein was involved in production of cytokines in response to TLR4 and TLR9 stimulation, but NF-κB activation was not completely deficient in IRAK2 knockout cells (67, 144). Finally, study of TLR and IL-1R responses in murine macrophages lacking both IRAK1 and IRAK2 demonstrated that these proteins are somewhat redundant in those cells, albeit with key differences in their behavior (67).

The crystal structure of the myddosome reveals a helical architecture containing multiple copies of MyD88, IRAK4 and IRAK2. While this model does not include IRAK1 due technical limitations (the authors were unable to express IRAK1), the IRAK2 residues required for interaction with IRAK4 in the myddosome are also found in IRAK1 (15). This, in addition to immunoprecipitation data showing that IRAK1 is found in the myddosome when IRAK4 is present, strongly suggests that either IRAK1 or IRAK2 are recruited to this SMOC and can play similar roles (41, 42, 103).

The history of the discoveries of these IRAKs, as well as the myddosome structure, suggests that these proteins are somewhat redundant, and only double knockout cells show a clear phenotype. This is, however, an oversimplification. The roles played by IRAK1 and IRAK2 on cytokine production can be context dependent, varying according to species, cell type, duration of stimuli, etc. Additionally, other biologically relevant activities such as inflammasome activation and metabolism regulation appears to employ one IRAK and not the other.

### 5.1 IRAK2 is required for sustained signaling in murine macrophages

In murine macrophages, IRAK1 and IRAK2 show redundancy at early time-points. Knockout or knockdown of either IRAKs in those cells fail to decrease NF-κB and MAPK activation, while IRAK1/IRAK2 double knockout macrophages are highly deficient. Despite this early redundancy, IRAK2 knockout cells are critically impaired in inflammatory cytokine production while IRAK1 knockouts behave like wild type controls (67, 77, 87). One possible explanation for this phenomenon is that IRAK1 is degraded after stimulation, making IRAK2 essential for sustained signaling. This hypothesis is further corroborated by the existence of IRAK1b, a splice variant with prolonged stability and capable of sustaining NF-κB activation in human cells (145). This partial redundancy, however, appears to be context dependent. For instance, *Irak1^-/-^* mouse embryonic fibroblasts fail to activate NF-κB in response to IL-1β or IL-18 (43), whereas *Irak1^-/-^* macrophages activate NF-κB unimpaired in response to a variety of TLR agonists (67, 77). In humans, IRAK1-deficient macrophages are greatly deficient in TNF production, while IRAK2 deficiency has little to no impact, opposite to what is observed in mouse macrophages (136).

### 5.2 IRAK1 activates IRFs 1, 5 and 7

The activation of transcription factors and control of gene transcription are possibly the most important responses mediated by the myddosome. Although NF-κB activation is often used as surrogate for myddosome activity, this is not the only family of transcription factors controlled by this SMOC. The IRF family, in particular IRFs 1, 5 and 7, are also activated downstream MyD88 in a variety of conditions (2). As discussed above, NF-κB activation often displays redundancy regarding the use of IRAK1 and IRAK2. This redundancy, however, is not observed on the activation of IRF proteins.

In plasmacytoid dendritic cells (pDCs), activation of TLRs leads to a MyD88-dependent production of interferon-α (IFN-α), tumor necrosis factor (TNF), and IL-12 (146), with the IFN-α output primarily regulated by the transcription factor IRF7 (37). Similar to what is observed in murine macrophages, IRAK1 knockout pDCs are not deficient in MAPK and NF-κB activation upon stimulation of TLRs 7 or 9. Accordingly, these *Irak1^-/-^* pDCs produced normal levels of TNF and IL-12. Surprisingly, the production of IFN-α was highly deficient in *Irak1^-/-^* pDCs (147). TLR stimulation of pDCs leads to physical interactions between MyD88, IRAK4, IRAK1, TRAF6 and IRF7 (36, 147). IRAK1 kinase activity is required for IRF7 activation, possibly by controlling IKK-α, responsible for IRF7 phosphorylation. This leads to IRF7 dimerization and nuclear translocation (34, 147–150). Contrary to NF-κB and MAPK activation, IRAK1, but not IRAK2, is responsible for IRF7 stimulation (84, 151). In fact, TLR9-stimulated IRAK2 knockout pDCs shows higher IFN-α production than WT controls, suggesting that this kinase inhibits IRF7 by an yet unknown mechanism (151).

In TLR9-stimulated macrophages and myeloid dendritic cells (mDCs), production of IFN-β is controlled by the transcription factor IRF1 (152). IRF1 activation requires physical interaction with the myddosome, and most likely involves phosphorylation mediated directly or indirectly by IRAK1 (152, 153). Although it is tempting to speculate that IRF1 activation is, like IRF5 and IRF7, a process that requires IRAK1 and not IRAK2, experimental evidence is still required to rule out the involvement of IRAK2 in this pathway.

IRF5 activation occurs downstream MyD88-dependent TLRs, with MyD88, TRAF6, and IRAK1 required for its activation (34, 35). This suggests that myddosome formation and physical interaction between IRF5 and its components is essential. Indeed, IRAK1:IRF5 interaction precedes IRF5 ubiquitination by TRAF6 and activation (34, 154). In addition to ubiquitination, IRF5 phosphorylation by IKK-β is required to its dimerization and nuclear translocation (39, 40). This activity appears to be mediated by IRAK1 exclusively, as inhibition of IRAK2 signaling by the poxvirus A52 protein failed to impact IRF5 activation (55, 84). The precise timing of all the sequential steps involved in IRF5 activation is unclear, and whether ubiquitination by TRAF6 occurs in physiological conditions remains an open question. Regardless, studies on human monocytes expressing physiological concentrations of IRAK1 and IRF5 also suggests that this transcription factor requires IRAK1, as loss of this IRAK antagonizes IRF5 signaling (155).

While IRFs 1 and 7 regulate the production of type I interferons (37, 38, 156), IRF5 is involved in the control of various pro-inflammatory cytokines (35). Indeed, the set of genes regulated by IRF5 and NF-κB show overlap, and physical interaction between IRF5 and NF-κB is a potential mechanism for gene regulation (157). How IRF5:NF-κB interactions occur and how this process is regulated remains poorly understood.

### 5.3 IRAK1 controls rapid inflammasome activation

Activation of the NLR family pyrin domain containing 3 (NLRP3) Inflammasome occurs in two phases. In the first phase, a signal primes the cell, enabling it to respond to a DAMP. In the second phase, recognition of a DAMP triggers the assembly of the inflammasome, a macromolecular structure responsible for triggering pyroptosis *via* caspase-1 activation and maturation of pro-IL-1β and pro-IL-18 (158). The priming phase includes both transcriptional and non-transcriptional responses, such as upregulation of key-components (pro-IL-1β and NLRP3 itself for example), and control of post-translational modifications that enables the inflammasome to respond.

It is well established that transcriptional priming in many situations involves the myddosome (and consequentially IRAKs) (2), but study of non-transcriptional priming of the NLRP3 inflammasome revealed an additional role for IRAK4 and IRAK1. IRAK1-deficient mouse macrophages fails to activate caspase-1, whereas IRAK2-deficient cells behave like wild type controls (23). This response is rapid, precedes transcriptional priming, and allows the NLRP3 inflammasome to quickly release pre-synthesized IL-18 in response to DAMPs (22, 23). While it is unclear how this happens, it is tempting to speculate that IRAK1 phosphorylates components of the inflammasome to fine-tune its assembly. Phosphorylation in the NLRP3 leucine rich repeat controls inflammasome assembly, and mutations on a key serine residue blocks non-transcriptional priming (159). This is further suggested by the observation that kinase-deficient IRAK1 and IRAK4 fail to prime the inflammasome, and physical interactions between IRAK1 and components of the inflammasome can be observed (23).

While IRAK1 appears to stimulate NLRP3 activity in the context of rapid inflammasome activation, it can also act as a negative regulator in other conditions. Upon TLR and IL-1R activation, IRAK1 is ubiquitinated by the ubiquitin ligase Pellino 2 (28, 32). Ubiquitinated IRAK1 cannot interact with NLRP3, which allows Pellino 2 to ubiquitinate and activate NLRP3. In IRAK1-deficient macrophages (or containing a kinase-dead IRAK1), NLRP3 ubiquitination occurs at a higher rate and inflammasome activation is increased. When macrophages are deficient for Pellino 2, however, interactions between IRAK1 and NLRP3 are enhanced, and inflammasome activation is deficient (160).

These seemingly contradictory reports present an incomplete view on how IRAK1, as well as other IRAKs, fine-tunes inflammasome priming and activation. Further investigation is required to understand the different roles that these kinases have on inflammasome activity.

### 5.4 IRAK2 inhibits oxidative metabolism

While this review has primarily focused on how IRAKs regulates cytokine production, a large amount of recent studies have highlighted another important aspect involved in innate immune responses: modulation of cell metabolism (161). We are only beginning to understand the links between IRAKs and metabolism, with recent studies suggesting that these kinases both regulate and are regulated by cell metabolism.

TLR activation in myeloid cells leads to rapid transition to aerobic glycolysis and increase in succinate concentration. Succinate then acts as a positive regulator of the transcription factor hypoxia inducible factor-1α (HIF-1α) which stimulates the expression of genes such as *IL1B* and *IRAKM* (162–164). HIF-1α also controls the transcription of microRNA-146a, which act as a negative regulator of pro-inflammatory gene expression by downregulating the expression of TRAF6 and IRAK1 (165, 166). Another important player in the rapid stimulation of glycolysis *via* TLRs is TBK1 (167). This protein kinase is known to mediate IRF3 activation downstream TRIF (168), but surprisingly, induction of glycolysis by TBK1 requires myddosome assembly and physical interaction with its components (25). It is presently unknown which IRAKs, if any, mediate myddosome-dependent TBK1 activation.

A more direct link between IRAKs and metabolism comes from the observation that IL-1β inhibits mitochondrial oxidative phosphorylation in adipocytes (26). Upon IL-1R activation, MyD88, IRAK4 and IRAK2 translocate to the mitochondrial outer membrane. From there, IRAK2 further translocates into the mitochondrial inner membrane and inner membrane space *via* the translocators TOM20 and TIMM50. IRAK2 then inhibits oxidative phosphorylation by disrupting the interaction between prohibitin (PHB) and optic atrophy protein 1 (OPA1), key in the formation of the mitochondrial respiratory chain complex. Importantly, myddosome formation triggers IRAK2 auto-phosphorylation at residues S134 and T140 (24, 79), which are required for IRAK2 translocation and interaction with PHB-OPA1. Interestingly, in adipocytes IRAK1 deficiency did not disrupt oxidative phosphorylation, and IRAK2 deficiency did not affect pro-inflammatory gene expression, further evidence that IRAK1 and IRAK2 play different role in different cell types (26).

## 6 IRAK mutations in human diseases

IRAK proteins are the main actors coordinating myddosome activity and play essential roles in the innate immune response in mammals. However, important details of their biology differ between humans and other animals. For instance, the kinase activity of IRAK4 does not impair the responses in human macrophages (136), suggestive that in humans this kinase might be involved in other pathways. Similarly, IRAK1 has a more prominent role in humans, while IRAK2 appears to be redundant (136). As such, mutations affecting IRAK1 and IRAK4 can have dramatic clinical impact in humans.

IRAK4 mutations are clinically relevant and life-threatening in childhood, with a mortality rate estimated at 38% (169). Most of the studied IRAK4 disorders are autosomal recessive, often caused by point mutations introducing an early stop codon, leading to virtually absent IRAK4 expression or production of a non-functional protein (49, 50, 169, 170). This results in high susceptibility to pyogenic bacterial infections, specially *Staphylococcus aureus*, *Streptococcus pneumoniae*, and *Pseudomonas aeruginosa* (169). However, cases have been reported linking IRAK4 mutations to susceptibility to other bacterial agents such as *Salmonella* serogroup C1, *Listeria monocytogenes, Shigella sonnei* and non-bacterial agents such as herpesvirus (51, 171–173).

Little is known about IRAK1 loss-of-function mutations in humans, but evidence suggests that such mutations potentially lead to increased susceptibility to infections. For instance, investigation of a patient who died as result of pulmonary infection at the age of 7 months revealed complete IRAK1 deletion. This deletion may cause a X-linked recessive disorder with severely impaired TLR responses in fibroblasts, but not in peripheral blood mononuclear cells (54). IRAK-M has several anti-inflammatory activities, and single nucleotide polymorphisms in the *IRAK3* gene were associated with higher susceptibility to asthma, likely due deficient anti-inflammatory responses in airway epithelial cells (52).

IRAK1 gain-of-function mutations are more common and may lead to enhanced sepsis-induced injury due increased pro-inflammatory responses (174). Interestingly, various reports suggest that IRAK1 mutations causing enhanced protein expression or spontaneous activation act as an oncogene and are involved in development of various cancers (53, 175–178). Similarly, IRAK4 gain-of-function mutations are associated with cancers and linked to poor prognosis and resistance to chemotherapy (179–181).

## 7 Conclusion and perspectives

Since the discovery that IL-1R signaling requires a protein kinase (41, 42), many aspects of the IRAK family were studied. After decades of efforts, we understand in broad strokes their biology, but many of the finer details remain elusive. For instance, the myddosome is assembled and signal downstream IL-1Rs and most TLRs, which is both a blessing and a curse: lessons learned from a specific TLR or IL-1R are applied to all other receptors, but important details of specific receptors might be missed.

The myddosome can recruit either IRAK1 or IRAK2 to transduce signals (15), and different cell types appear to employ one IRAK preferentially. What causes the preferential use of one over the other is largely unknown. Study of cell-specific responses may lead to better understanding on how these kinases affect and are affected by different microenvironments. Similarly, human and mouse cells often diverge regarding the use of IRAKs 1 and 2, but the reasons for these species-specific phenotypes are unknown.

The links between pattern recognition receptors and metabolism are only beginning to be understood, but it is clear that they deeply influence each other (161). For example, myddosome assembly upon TLR activation triggers glycolysis *via* TBK1 (25). It is unclear whether any IRAKs are important in this pathway, but since TBK1 interacts with the myddosome it is tempting to speculate that IRAKs are upstream TBK1. Another report suggests that IRAK2 directly inhibits oxidative phosphorylation in IL-1R-stimulated adipocytes (26). The study of how the myddosome and IRAKs are involved immunometabolism is still in its early days and remain an exciting prospect.

The observation that inhibition of IRAK4 kinase activity has less impact in human than in murine macrophages is particularly interesting (136), as it suggests that in humans this kinase might be involved in other pathways, and only the IRAK4 scaffold is required for TLR signaling. This scaffold, and potentially other IRAKs, might also be involved in TRAF6 activation mediated by the putative triffosome by yet unknown mechanisms (77). A deeper understanding of the differences between IRAK4 kinase and scaffold activities is likely to be clinically relevant, as both IRAK4 inhibitors and degraders are currently in clinical trials (137) and can potentially impact the treatment of a wide range of inflammatory and autoimmune diseases.

## Author contributions

MP wrote the manuscript. RG revised the manuscript. All authors contributed to the article and approved the submitted version.
